# Psoas Abscess Precipitated by an Uncommon Pathogen: Pasteurella multocida

**DOI:** 10.7759/cureus.39376

**Published:** 2023-05-23

**Authors:** Scott C Everett, Andrew L Alejo, Joseph P Myers

**Affiliations:** 1 College of Medicine, Northeast Ohio Medical University, Rootstown, USA; 2 Department of Medicine, Summa Health, Akron, USA

**Keywords:** percutaneous drainage, infection, case report, psoas abscess, pasteurella multocida

## Abstract

A psoas abscess is a rare infection; it is an accumulation of purulent material within the psoas muscle. Common pathogens include *Staphylococcus aureus*, streptococci, *Escherichia coli*, and other enteric Gram-negative bacilli and anaerobes. These abscesses are thought to occur by either hematogenous spread, contiguous spread from adjacent organs, trauma, or local inoculation. *Pasteurella multocida *is a pathogen that usually infects a patient via a bite or scratch from dogs or cats and causes cellulitis at the site of the injury. *Pasteurella multocida *may also cause infection by the colonization of human respiratory and gastrointestinal (GI) tracts with spontaneous bacteremia seeding remote organs by the bacterial translocation process. *Pasteurella multocida* is highly susceptible to penicillins, cephalosporins, and other antibiotics. However, psoas abscesses usually require a drainage procedure as well as an extended course of antibiotics. We present a patient presenting with a psoas abscess due to *P. multocida*,* *an uncommon presentation of infection by this bacterium.

## Introduction

A psoas abscess is a collection of purulent material within the iliopsoas compartment. The psoas muscle originates at the bodies of the vertebrae from T12 to L4 and inserts at the lesser trochanter of the femur, after joining the iliacus muscle to form the iliopsoas muscle [1]. The main function of this muscle is to flex and externally rotate the hip joint. A psoas abscess is most frequently seen in children and young adults; however, it is an unusual infection in adults with a reported incidence worldwide of 0.4 cases per 100,000 individuals [2]. These abscesses are believed to arise from either hematogenous spread from distant sites, trauma to the hip or lumbar areas, or contiguous spread from adjacent structures (sigmoid colon, appendix, ureters, abdominal aorta, kidneys, and pancreas) [3]. It may also arise as a complication of epidural anesthesia [4].

Common pathogens include *Staphylococcus aureus*, both methicillin-susceptible and methicillin-resistant, streptococci, *Escherichia coli*, and other enteric aerobic and anaerobic Gram-negative bacilli. Risk factors for developing a psoas abscess include diabetes mellitus, alcoholism, acquired immunodeficiency syndrome, immunosuppression, injection drug use, Crohn’s disease, and malnutrition [5]. Atypical pathogens such as *Pasteurella multocida* have been rarely reported in the literature as a cause of psoas abscesses [2,5-7]. *Pasteurella multocida* is most commonly introduced into the body following a bite or a scratch from domestic pets such as dogs or cats and mainly causes cellulitis at the site of the injury. However, *P. multocida* may also colonize human respiratory and gastrointestinal (GI) tracts from animal non-bite contact and subsequently cause end-organ infection via translocation of the organism from the respiratory or GI tracts. First-line therapy is often with penicillin such as ampicillin-sulbactam or piperacillin-tazobactam, but the organism is usually susceptible to cephalosporins, fluoroquinolones, and trimethoprim-sulfamethoxazole [6]. In this case, we present a 65-year-old female who developed a psoas abscess caused by *Pasteurella multocida*.

## Case presentation

A 65-year-old Caucasian female with a past medical history of hypertension, hyperlipidemia, and hypothyroidism was evaluated in the emergency department (ED) after sustaining multiple mechanical falls over several days at her group home. Vitals included a blood pressure of 110/82 mmHg, respiratory rate of 18/minute, pulse of 112/minute, and a temperature of 36.3°C. Systemic examination was normal, including a normal abdominal examination, except for tenderness to palpation of the left hip and buttocks. At this time, she had a white blood cell count of 30,200/µL (reference range: 4,500-11,000/µL), hemoglobin of 5.8 g/dL (reference range: 12-16 g/dL), mean corpuscular volume of 74.6 fL (reference range: 80-100 fL), and platelet count of 853,000/µL (reference range: 150,000-450,000/µL). She received intravenous (IV) fluid resuscitation, empiric IV vancomycin and piperacillin-tazobactam, and two units of packed red blood cells. A fecal occult blood test was positive, and a chest X-ray was notable for a mass-like consolidation in the right lower lobe. She was continued on IV antibiotics and admitted for further evaluation.

**Figure 1:**
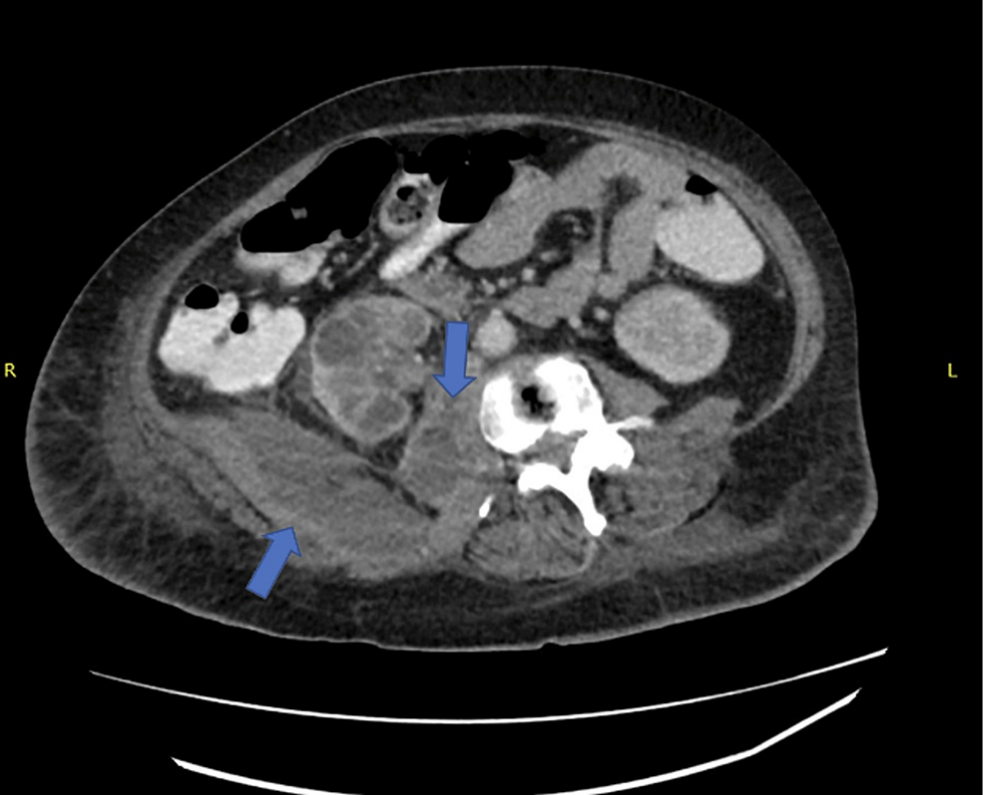
Initial computed tomography demonstrating evidence of a psoas abscess with a large fluid collection around the psoas muscle and retroperitoneal tissues (arrows)

Once admitted, she complained of left hip pain and mild right-sided abdominal pain. She had not noticed any bright red blood per rectum or gross blood in her stool recently. She endorsed arthralgias, decreased appetite, unintentional weight loss, and tenderness to the left hip and right lower quadrant upon palpation. She mentioned that her only animal contact was the petting of some feral cats in the backyard of her group home. There was no history of cat, dog, or other animal bites or scratches. She did endorse a cat dander allergy as well as owning many cats. She stated that she had no recent history of travel as well. Infectious disease was consulted for further evaluation of her leukocytosis, and the workup consisted of a viral upper respiratory panel, which came back negative, and a computed tomography (CT) of the abdomen/pelvis. The CT revealed fluid collections involving the psoas muscle and right retroperitoneal soft tissues (Figure 1). CT of the abdomen/pelvis also revealed a stellate mesenteric mass tethered to the cecum and adjacent soft tissues including the uterus and right adnexa suggestive of a carcinoid tumor for which surgery was consulted.

**Figure 2:**
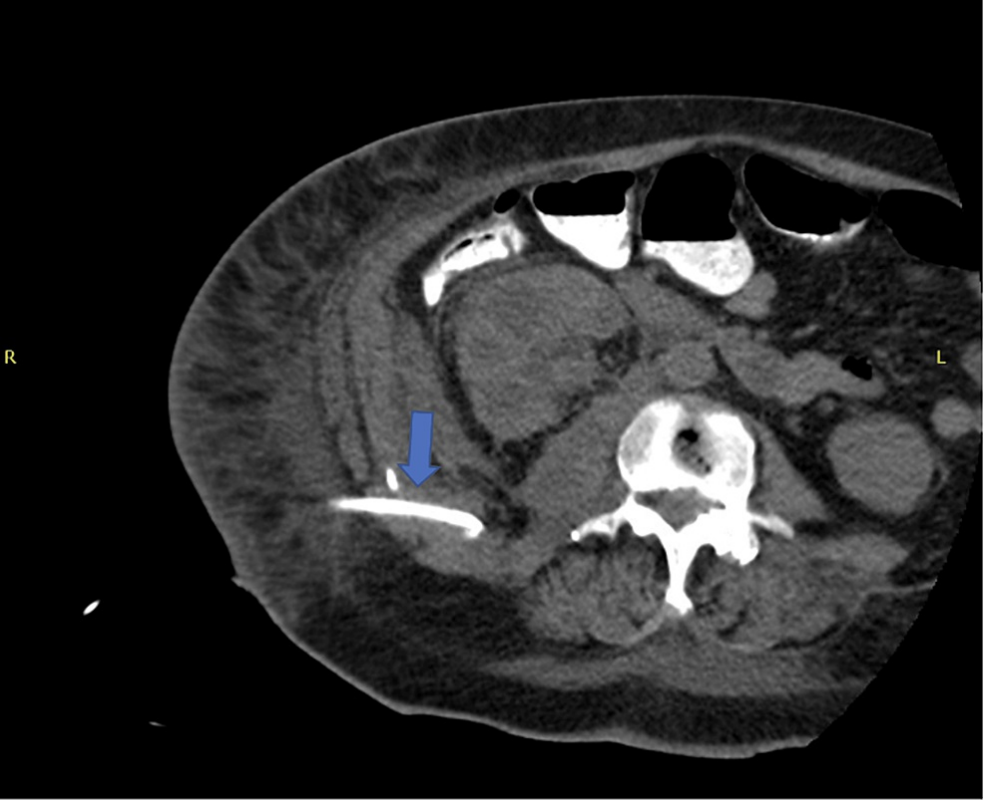
Imaging during computed tomography-guided drainage shows catheter placement in the center of the abscess, with a significant decrease in abscess volume (arrow)

**Figure 3:**
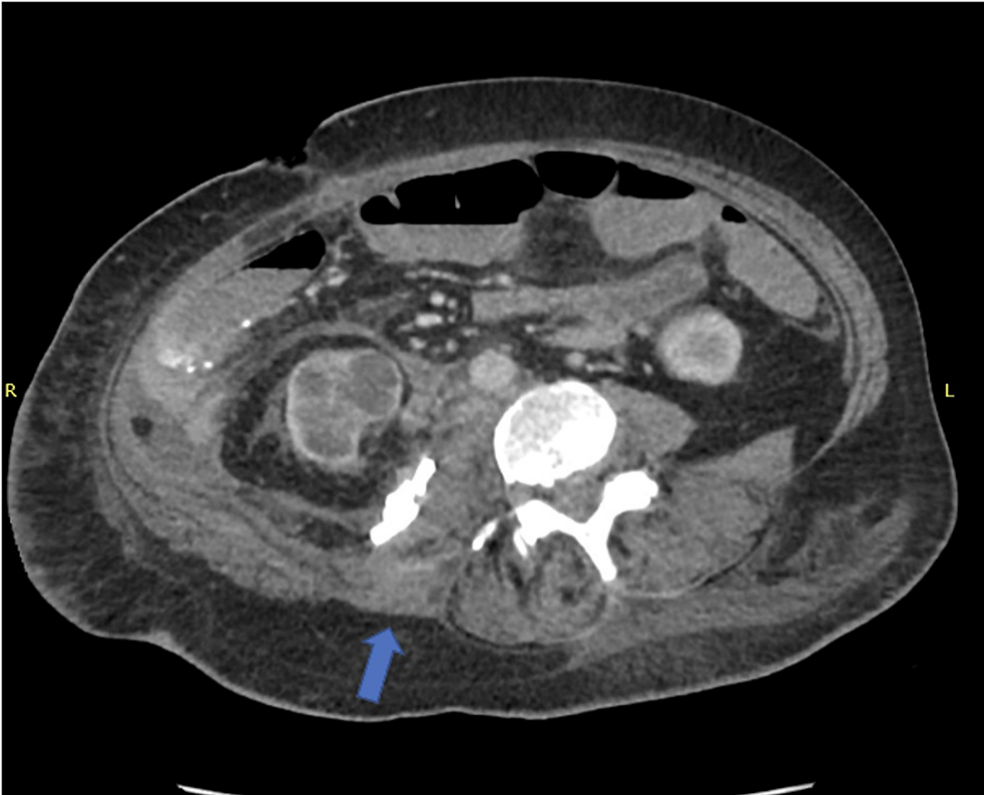
Imaging 10 days after drain placement shows minimal fluid in the area surrounding the psoas muscle and the adjacent retroperitoneal tissues (arrow)

On hospital day 3, the patient underwent CT-guided retroperitoneal abscess drainage yielding 230 mL of thick brown fluid with eventual isolation of pure growth of *Pasteurella multocida* with antimicrobial susceptibility testing (Figure 2). The patient was continued on piperacillin-tazobactam based on the results of the susceptibility testing, for four weeks, most of which was completed after transfer to a long-term acute care hospital. On hospital day 8, the patient underwent a right hemicolectomy with ileocolic anastomosis with placement of a wound vac (adjunct to promote healing) for the removal of the mass found on CT. The gross appearance of the removed mass appeared to be cancerous, and upon histopathological analysis of the mass, it was found to be a carcinoid tumor. Repeat CT imaging before discharge showed resolution of the psoas abscess (Figure 3). Blood cultures taken from day 1 still had no growth to date, and the patient was eventually discharged back to her previous facility with a referral for follow-up by her primary care physician for reevaluation of symptoms.

## Discussion

Patients with a psoas abscess often have vague symptoms that may present subacutely. Typical symptoms include fever, low back or hip pain that may radiate to the groin, a lump, and malaise [1]. Delayed diagnosis of a psoas abscess can lead to anemia, weight loss, or anorexia. Psoas abscess should be considered as part of the evaluation for fever of unknown origin. The current best practice to diagnose a psoas abscess is using computed tomography (CT), which can localize and demonstrate the extent of the abscess [8]. CT of the abdomen and pelvis along with elevated inflammatory markers and blood and abscess cultures help guide therapy. Percutaneous drainage of a psoas abscess is critical to decreasing the bacterial load and allows the clinician to identify a specific pathogen allowing for pathogen-guided antibiotic microbial therapy. After a diagnostic aspiration under CT guidance, treatment with broad-spectrum antibiotics should be initiated to treat the most common pathogens causing psoas abscesses.

Psoas abscesses are unusually rare and compromise 6% of intra-abdominal abscesses [9]. Hematogenous spread or spread from an adjacent organ is most frequently reported; however, diseases of the gastrointestinal tract (diverticulitis, appendicitis, and Crohn’s disease) increase the patient’s susceptibility to developing these abscesses. Although she did not have any metastasis of her carcinoid tumor, which could then develop into a carcinoid syndrome, the presence of this tumor within her gastrointestinal tract may have promoted the development of the abscess via local inflammation. Musculoskeletal, genitourinary, and vascular infections are also frequent causes. Clinical presentations of psoas abscesses are often variable and vague, similar to how our patient presented. The classic triad of fever, groin or back pain, and a lump is only present in approximately 30% of patients, allowing for a rapid diagnosis [6]. Laboratory studies commonly show leukocytosis, thrombocytosis, and elevated inflammatory markers.

In our patient, both laboratory values and imaging were helpful in determining the patient’s treatment. Her exposure to feral cats in the backyard of her group home was most likely the source of the infection; however, it was unable to be confirmed given her history. Cultures eventually revealed *Pasteurella multocida *as the causative microorganism. The patient’s complaints and physical findings were inadequate to make a diagnosis of psoas abscess. The psoas sign (abdominal pain with hip extension) was not performed in this patient because there was a concern for a hip fracture. Her CT scan, the gold-standard diagnostic procedure for psoas abscess, was successfully able to demonstrate her psoas abscess. The primary treatment was CT-guided percutaneous drainage followed by extended parenteral antimicrobial therapy.

Psoas abscess due to *Pasteurella multocida* is rare. Only four other cases of psoas abscess caused by *Pasteurella multocida* have been reported. The most recent report of *P. multocida *psoas abscess was that of an elderly female patient with fever, right groin pain, and malaise with exposure to cats [7]. CT scan of the abdomen and pelvis was the test that allowed for the visualization of the abscess, leading to percutaneous drainage and organism-specific antimicrobial therapy. Another case by Steiner et al. [10] showcased a nine-year-old girl with a history of meningomyelocele and a kidney transplant presenting with progressive gait disturbance. She was treated similarly and successfully recovered from her abscess.

## Conclusions

We report a case of psoas abscess caused by *Pasteurella multocida*, an extremely rare etiologic agent for this disease. Our patient, with known cat exposure but no history of cat or dog scratches, bites, or dermatologic manifestations such as cellulitis, developed her abscess that was successfully identified on a CT scan and treated with percutaneous drainage and intravenous antibiotics. Percutaneous drainage is critical for both primary treatment (source control) and identification of causative microorganism(s).
